# Effects of Ursolic Acid and its Analogues on Soybean 15-Lipoxygenase Activity and the Proliferation Rate of A human Gastric Tumour Cell Line

**DOI:** 10.1155/S0962935194000244

**Published:** 1994

**Authors:** D. Es-Saady, A. Najid, A. Simon, Y. Denizot, A. J. Chulia, C. Delage

**Affiliations:** 1 Equipe ‘Biomolécules’ Faculté de Pharmacie 2 rue Dr Marcland Limoges 87025 France; 2 Laboratoire Immuno-Hémato-Biochimie (IHB) Pôle Recherche GCA Groupe Guerbet Chasse sur Rhône 38690 France; 3 Laboratoire d'Hématologie Expérimentale Faculté de Médecine 2 rue Dr Marcland Limoges 87025 France

## Abstract

The authors have previously isolated and purified ursolic acid from heather flowers (*Calluna vulgarts*). This terpene was found to inhibit HL-60 leukaemic cell proliferation and arachidonic acid oxidative metabolism in various cell species. The effects of ursolic acid and its analogues on soybean 15-lipoxygenase activity and on the proliferation of a human gastric tumour cell line (HGT), have been assessed. These triterpenes inhibited soybean 15-lipoxygenase at its optimal activity (pH 9). The proliferation ofHGT was decreased in a dose-dependent manner. At 20 μM the rank order is: ursolic acid > uvaol > oleanolic acid > methyl ursolate. The carboxylic group at the C_28_ position of ursolic acid appears to be implicated in the inhibition of both lipoxygenase activity and cell proliferation. Thus methylation of this group decreases these two inhibitory properties. Oleanolic acid, which differs by the position of one methyl group (C_20_ instead of C_19_) is less inhibitory than ursolic acid. The lipophilicity of the terpene is also implicated since uvaol appears to be more inhibitory than methyl ursolate.


Mediators of Inflammation 3, 181–184 (1994)

### Effects of ursolic acid and its analogues on soybean 15-lipoxygenase activity and the proliferation rate of a human gastric tumour cell line

D. Es-Saady,[1] A. Najid,[2] A. Simon,[1]

Y. Denizot,[3] A. J. Chulia[1] and C. Delage[1,CA]

[1]Equipe 'Biomolécules', Faculté de Pharmacie, 2 rue Dr Marcland, 87025 Limoges, France; [2]Laboratoire Immuno-Hémato-Biochimie (IHB), Pôle Recherche GCA, Groupe Guerbet, 38690 Chasse sur Rhône, France; [3]Laboratoire d'Hématologie Expérimentale, Faculté de Médecine, 2 rue Dr Marcland, 87025 Limoges, France

[CA] Corresponding Author

**The authors have previously isolated and purified ursolic acid from heather flowers (*Calluna vulgaris*). This terpene was found to inhibit HL-60 leukaemic cell proliferation and arachidonic acid oxidative metabolism in various cell species. The effects of ursolic acid and its analogues on soybean 15-lipoxygenase activity and on the proliferation of a human gastric tumour cell line (HGT), have been assessed. These triterpenes inhibited soybean 15-lipoxygenase at its optimal activity (pH 9). The proliferation of HGT was decreased in a dose-dependent manner. At 20 μM the rank order is: ursolic acid > uvaol > oleanolic acid > methyl ursolate. The carboxylic group at the $C_{28}$ position of ursolic acid appears to be implicated in the inhibition of both lipoxygenase activity and cell proliferation. Thus methylation of this group decreases these two inhibitory properties. Oleanolic acid, which differs by the position of one methyl group ($C_{20}$ instead of $C_{19}$) is less inhibitory than ursolic acid. The lipophilicity of the terpene is also implicated since uvaol appears to be more inhibitory than methyl ursolate.**

**Key words:** HGT gastric cancer cell line, Lipoxygenase inhibitor, Proliferation, Ursolic acid

### Introduction

Arachidonic acid can be oxygenated by two distinct pathways, cyclooxygenase and lipoxygenase, which differ not only in their products but also in their susceptibility to regulation by biological and pharmacological agents.[1] Additionally, a third pathway for arachidonic acid oxygenation, via NADPH-dependent cytochrome P-450 monooxygenases (epoxygenases), has been described recently in various tissues and cells.[2] The lipoxygenase pathway converts arachidonic acid to a family of hydroxy (peroxy)eicosatetraenoic acids (H(P)ETEs) and to a series of more complex metabolites termed leukotrienes, lipoxines and hepoxylines.[2] The functional properties of the lipoxygenase products have not been elucidated completely. However, it is clear that the lipoxygenase pathway generates the release of extracellular lipid mediators of inflammation such as leukotrienes $B_4$ ($LTB_4$), $LTC_4$, $LTD_4$ and $LTE_4$.[3] Several studies have suggested a role for intracellular mediators in the formation of lipoxygenase products.[4,5]

In an effort to search for new anti-inflammatory and/or new anti-proliferative natural compounds, the authors have used the inhibition test of these enzymes to screen several heather flower extracts of *Calluna vulgaris*. This plant is mainly used in folk medicine as extracts, especially in the treatment of inflammatory diseases.[6] An aqueous extract of *Calluna vulgaris* (CVE) shows inhibition specifically of arachidonate 5-lipoxygenase and potent anti-proliferative effects on human leukaemia HL-60 cells.[7] In acetone–CVE, ursolic acid has been shown as the agent responsible for inhibition of lipoxygenase activity. Ursolic acid was found to be an inhibitor of both potato tuber 5-lipoxygenase and soybean 15-lipoxygenase.[8] Ursolic acid also inhibits arachidonic acid metabolism in platelets, mouse peritoneal macrophages and differentiated HL-60 leukaemic cells.[9] Subsequently, the X-ray crystallographic structure of the molecule was determined and it was shown that the compound adopts a chair conformation.[10]

In this paper the effects of ursolic acid and its analogues (uvaol, oleanolic acid and methyl ursolate) on both lipoxygenase activity and proliferation of a human gastric tumour cell line (HGT)[11] were studied in order to obtain more information on the chemical reactions of ursolic acid that are implicated in its pharmacological action.

### Materials and Methods

*Chemicals:* Ursolic acid was either obtained from Sigma or isolated from *Calluna vulgaris* as described previously.[8] Uvaol and oleanolic acid were obtained from Sigma. Methyl ursolate was prepared by methylation of ursolic acid with diazomethane.[12]

© 1994 Rapid Communications of Oxford Ltd

*Lipoxygenase assay:* The standard assay mixture contained the enzyme soybean 15-lipoxygenase (from Sigma) and the reaction was started by addition of linoleic acid (Sigma). Inhibition experiments were carried out by measuring the loss of activity of 15-lipoxygenase (0.11 μM) in the presence of various concentrations of triterpene. Lipoxygenase activity was determined spectrophotometrically by monitoring the absorbance at 234 nm of 13-(*S*)-hydroperoxy-*cis*-9-*trans*-11-octadecadienoic acid (13(*S*)-HPODE) ($E_{max}$ = 25 000 $M^{-1}cm^{-1}$) formed from linoleic acid (71 μM), at 20°C in 1 ml of 0.2 M borate buffer (pH 9).

*Cell culture:* HGT cells at a concentration of $2 \times 10^5$ were grown (in triplicate) in 6-well culture plates (Nunc) in 2 ml of Dulbecco's modified Eagle's medium (Gibco) supplemented with 10% heat inactivated foetal calf serum (Gibco), penicillin (100 U/ml) and streptomycin (100 μg/ml) (Gibco) at 37°C in a humidified atmosphere containing 5% $CO_2$ in air. Drug solutions were added to cultures at day 1. At 24 h intervals, the viability of cells was determined as described below.

*Growth experiments:* Ursolic acid and its analogues were dissolved in DMSO (Merck). Stock solutions were prepared at 10 mM and stored at 4°C. Control culture received the same quantity of DMSO only. Cell counts and viable cells were assessed by trypan blue exclusion.

*MTT assay:* The MTT colorimetric assay was carried out as described initially by Mosmann.[13] This test is based upon the selective ability of living cells to reduce the yellow soluble salt, MTT (3-(4,5-dimethylthiazol-2-yl)-2, 5-diphenyl tetrazolium bromide) (Sigma), to a purple–blue insoluble formazan precipitate. Experiments were performed in triplicate in 6-well culture plates (Nunc). MTT was dissolved in phosphate buffered saline (PBS) at 5 mg/ml. After 24 h incubation of HGT cells with ursolic acid and its analogues, stock MTT solution (200 μl per 2 ml medium) was added and plates were incubated at 37°C for 4 h. Then sodium dodecyl sulphate (SDS) (10% in HCl, 0.01 M) was added and the amount of coloured formazan metabolite formed was determined by absorbance at 550–690 nm.

*Plating efficiency:* Cells were plated into wells containing 2 ml of growth medium alone or in the presence of drugs (20 μM). After 24 h, cells were washed with HBSS and were removed from wells with 0.5 ml of trypsin/EDTA (Gibco). Adherent cells were counted using a haemocytometer.

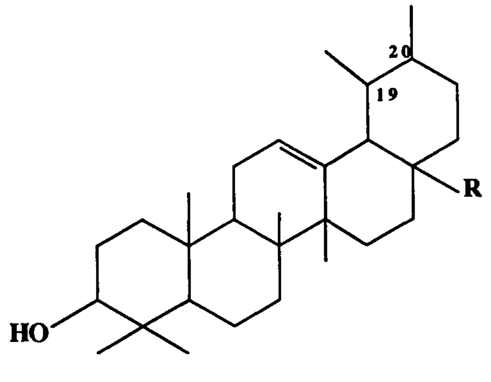

**R = COOH: ursolic acid**

**R = $CH_2OH$: uvaol**

**R = $COOCH_3$: methyl ursolate**

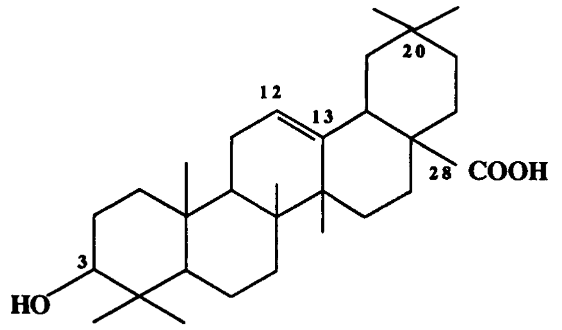

**oleanolic acid**

FIG. 1. Formulae of ursolic acid and analogues.

### Results

The chemical structures of ursolic acid and its analogues uvaol, oleanolic acid and methyl ursolate are shown in Fig. 1. The effects of the four compounds on soybean 15-lipoxygenase activity are shown in Fig. 2. The best inhibitors of soybean 15-lipoxygenase activity were ursolic acid and oleanolic acid, with a $IC_{50}$ values of 0.175 mM and 0.265 mM, respectively.

All the tested drugs (1 to 30 μM) had no effect on cell viability after 1 h and 6 h of incubation. Table 1 shows that ursolic acid has more effect on plating efficiency than does either uvaol or oleanolic acid. In contrast, its methyl ester had no effect on plating efficiency.

Figure 3 shows the dose-dependent inhibitory effect of ursolic acid and its analogues after 5 days of HGT cell proliferation. Proliferation of HGT cells was decreased by ursolic acid, with an $IC_{50}$ of 20 μM. In contrast, uvaol, oleanolic acid and methyl ursolate had no significant effect. Figure 4 shows the time-dependent inhibition of HGT cell proliferation using 20 μM ursolic acid and its analogues. Although ursolic acid exhibits a marked inhibitory effect, methyl ursolate is almost devoid of effect.

### Discussion

Lipoxygenase dependent growth has been reported for various malignant cell lines such as

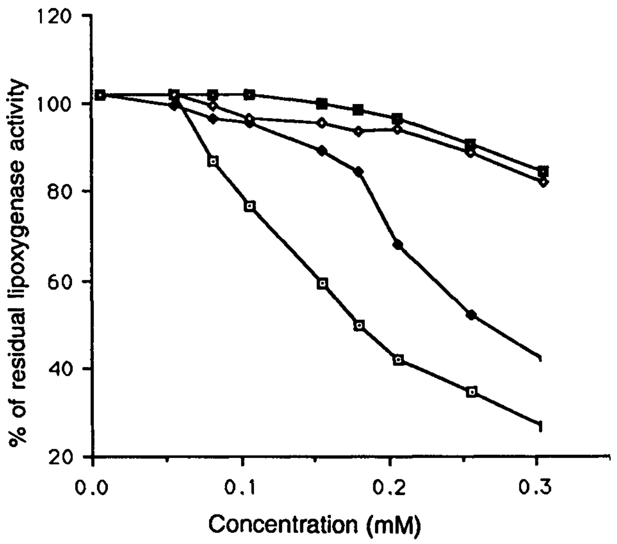

FIG. 2. Effects of ursolic acid and analogues on *in vitro* soybean 15-lipoxygenase activity (pH 9). Results are expressed in % of residual activity. ⊡, ursolic acid; ◆, oleanolic acid; ◇, methyl ursolate; ■, uvaol.

**Table 1.** Percentage of plating efficiency of HGT cells in the presence of ursolic acid and its analogues. Values are averages ± S.D. from two independent experiments, each done in triplicate ($n = 6$)

| Additions to media | Plating efficiency (%) |
|---|---|
| None | 90 ± 10 |
| Ursolic acid (20 μM) | 11.7 ± 8.3 |
| Uvaol (20 μM) | 45.6 ± 7.3 |
| Oleanolic acid (20 μM) | 85.5 ± 6.5 |
| Methyl ursolate (20 μM) | 92.1 ± 8 |

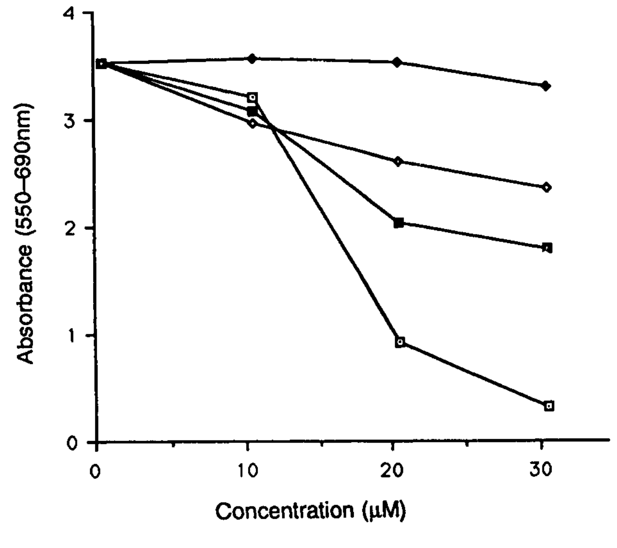

FIG. 3. Concentration-dependent inhibition of proliferation of HGT cells by ursolic acid and analogues, after 5 days of the growth. Results represent the mean of three independent experiments in triplicate. S.D. is always less than 5% in all cases. ⊡, ursolic acid; ■, uvaol; ◇, oleanolic acid; ◆, methyl ursolate.

neuroblastoma,[14] mouse melanoma[15] and MCF-7 human breast cancer cells.[16] Previous data have reported that the lipoxygenase inhibitor BW 755C suppressed the proliferation of HGT cells in a concentration dependent manner.[17] The authors have

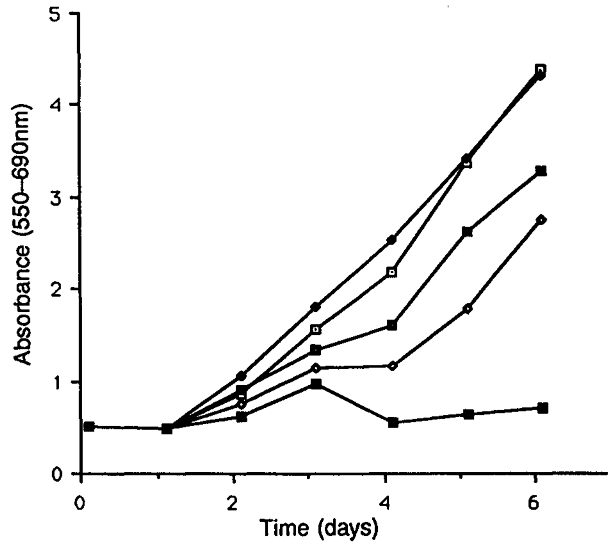

FIG. 4. Time-dependent inhibition of proliferation of HGT cells by ursolic acid and its analogues, at 20 μM. Results represent the mean of two independent experiments in triplicate. S.D. is always less than 5% in all cases. ■, ursolic acid; ◇, uvaol; ■, oleanolic acid; ⊡, control; ◆, methyl ursolate.

studied the effects of ursolic acid and its analogues on the proliferation of HGT cells and on the *in vitro* activity of soybean 15-lipoxygenase.

Results of the *in vitro* soybean 15-lipoxygenase assay show that the carboxylic group at position of $C_{28}$ ursolic acid is implicated in the inhibition of both lipoxygenase activity and cell proliferation. Methylation of this group eliminates these two inhibitory properties. The structural character of ursolic acid is also important for its inhibitory effects, since oleanolic acid, which differs from ursolic acid only in the position of one methyl group (at $C_{20}$ instead of $C_{19}$), is less inhibitory than ursolic acid. HGT cell proliferation is less sensitive to ursolic acid ($IC_{50}$ = 20 μM) than is HL-60 proliferation. Previous data reported that ursolic acid inhibited HL-60 DNA synthesis with an $IC_{50}$ value of 1 μM.[8]

Ursolic acid exhibits an effect on HGT cell proliferation at concentrations which do not directly inhibit 15-lipoxygenase activity. Furthermore, oleanolic acid, which inhibits soybean 15-lipoxygenase with an $IC_{50}$ of 0.265 mM, has only a small effect at 20 μM on HGT cell proliferation. Together, these data suggest that ursolic acid may act at this concentration (20 μM) by additional mechanisms other than just direct lipoxygenase inhibition. Another hypothesis is that the effect on tumour cells is a more sensitive test than the assay for lipoxygenase inhibition. Finally, the lipophilicity of the terpenes is also implicated in the effect of ursolic acid since uvaol is more inhibitory than methyl ursolate; this latter compound being more lipophilic than uvaol.

Ursolic acid probably acts on physical and functional properties of cell membranes and may be an additional class of membrane active agents with potential anti-cancer activity. It may be a pleiotropic membrane active agent that seeps into the lipidic layers and affects multiple signal transduction pathways in mammalian cells. Clearly further experiments need to be performed to clarify these points.

### References

1. Needleman P, Turk J, Jakschik BA, Morrison A, Lefkowith JB. Arachidonic acid metabolism. *Ann Rev Biochem* 1986; **55**: 69–102.
2. Fitzpatrick FA, Murphy RC. Cytochrome P-450 metabolism of arachidonic acid: formation and biological actions of 'epoxygenase'-derived eicosanoids. *Pharmacol Rev* 1989; **40**: 229–241.
3. Rouzer CA, Scott WA, Hamill AL, Cohn ZA. Synthesis of leukotriene C and other arachidonic acid metabolites by mouse pulmonary macrophages. *J Exp Med* 1982; **155**: 720–733.
4. Elliott GR, De Meent DV. Human recombinant interleukin-1a enhances basal and arachidonic acid-stimulated synthesis of cyclooxygenase and lipoxygenase metabolites by human skin fibroblasts *in vitro*. *Int J Immunopathol Pharmacol* 1991; **4**: 67–74.
5. Svensson U, Holst E, Sundler R. Protein-kinase-C independent activation of arachidonate release and prostaglandin E2 formation in macrophages interacting with certain bacteria. *Eur J Biochem* 1991; **200**: 699–705.
6. Olechnowicz-stepien W, Rzadkowska-Bodalska H, Grimshaw J. Investigation on lipid fraction compounds of heather flowers (*Calluna vulgaris* L.). *Pol J Chem* 1982; **56**: 153–157.
7. Najid A, Simon A, Delage C, Chulia AJ, Rigaud M. A *Calluna vulgaris* extract 5-lipoxygenase inhibitor shows potent antiproliferative effects on human leukemia HL60 cells. *Eicosanoids* 1992; **5**: 45–51.
8. Simon A, Najid A, Chulia AJ, Delage C, Rigaud M. Inhibition of lipoxygenase and human leukemic HL-60 cells proliferation by ursolic acid isolated from heather flowers (*Calluna vulgaris*). *Biochim Biophys Acta* 1992; **1125**: 68–72.
9. Najid A, Simon A, Delage C, Chulia AJ, Rigaud M. Characterization of ursolic acid as a lipoxygenase and cyclooxygenase inhibitor using macrophages, platelets and differentiated HL60 leukemic cells. *FEBS Lett* 1992; **299**: 213–217.
10. Simon A, Delage C, Saux M, Chulia AJ, Najid A, Rigaud M. Structure of ursolic acid ethanol solvate. *Acta Cryst* 1992; **C48**: 628–632.
11. Laboisse CL, Augeron C, Couturier-Turpin MH, Gespach C, Cheret AM, Potet F. Characterization of a newly established human gastric cancer cell line HGT-1 bearing histamine H2-receptors. *Cancer Res* 1982; **42**: 1541–1548.
12. Schlenk H, Gellerman JL. Esterification of fatty acids with diazomethane on a small scale. *Anal Chem* 1960; **32**: 1412–1414.
13. Mosmann T. Rapid colorimetric assay for cellular growth and survival: application to proliferation and cytotoxicity assays. *J Immunol Methods* 1983; **65**: 55–63.
14. Werner EJ, Waltenga RW, Dubowy RL, Bonne S, Stuart MJ. Inhibition of human malignant neuroblastoma cell DNA synthesis by lipoxygenase metabolites of arachidonic acid. *Cancer Res* 1985; **451**: 561–563.
15. Honn KV, Dunn JR. Nafazatrom (Bay g6575) inhibition of tumor cell lipoxygenase activity and cellular proliferation. *FEBS Lett* 1982; **139**: 65–68.
16. Najid A, Beneytout JL, Tixier M. Cytotoxicity of arachidonic acid and of its lipoxygenase metabolite 5-hydroperoxyeicosatetranoic acid on human breast cancer MCF-7 cells in culture. *Cancer Letts* 1989; **46**: 137–141.
17. Denizot Y, Najid A, Rigaud M. Effects of eicosanoids metabolism inhibitors on growth of a human gastric tumor cell line (HGT). *Cancer Letters* 1993; **73**: 65–71.

**Received 7 January 1994; accepted in revised form 2 February 1994**
